# Identification via Numerical Computation of Transcriptional Determinants of a Cell Phenotype Decision Making

**DOI:** 10.3389/fgene.2019.00575

**Published:** 2019-06-21

**Authors:** Marilisa Cortesi, Alice Pasini, Simone Furini, Emanuele Giordano

**Affiliations:** ^1^Laboratory of Cellular and Molecular Engineering “S. Cavalcanti”, Department of Electrical, Electronic and Information Engineering “G. Marconi” (DEI), Alma Mater Studiorum—University of Bologna, Bologna, Italy; ^2^Department of Medical Biotechnologies, University of Siena, Siena, Italy; ^3^BioEngLab, Health Science and Technology, Interdepartmental Center for Industrial Research (HST-CIRI), Alma Mater Studiorum—University of Bologna, Bologna, Italy; ^4^Advanced Research Center on Electronic Systems (ARCES), Alma Mater Studiorum—University of Bologna, Bologna, Italy

**Keywords:** computational modeling, epithelial-mesenchymal transition, cell decision, boolean model, markov model

## Abstract

Complex cellular processes, such as phenotype decision making, are exceedingly difficult to analyze experimentally, due to the multiple-layer regulation of gene expression and the intercellular variability referred to as biological noise. Moreover, the heterogeneous experimental approaches used to investigate distinct macromolecular species, and their intrinsic differential time-scale dynamics, add further intricacy to the general picture of the physiological phenomenon. In this respect, a computational representation of the cellular functions of interest can be used to extract relevant information, being able to highlight meaningful active markers within the plethora of actors forming an active molecular network. The multiscale power of such an approach can also provide meaningful descriptions for both population and single-cell level events. To validate this paradigm a Boolean and a Markov model were combined to identify, in an objective and user-independent manner, a signature of genes recapitulating epithelial to mesenchymal transition *in-vitro*. The predictions of the model are in agreement with experimental data and revealed how the expression of specific molecular markers is related to distinct cell behaviors. The presented method strengthens the evidence of a role for computational representation of active molecular networks to gain insight into cellular physiology and as a general approach for integrating *in-silico*/*in-vitro* study of complex cell population dynamics to identify their most relevant drivers.

## 1. Introduction

The ability to evolve and adapt to changing environments is a fundamental characteristic of every cell (Magyar et al., 2007; Balázsi et al., 2011) that exploits nested feedback loops, multistable dynamics, and gene expression noise to generate complex regulatory networks (Elowitz et al., 2002; Brandman et al., 2005; Ray and Igoshin, 2010; Tiwari et al., 2010). These processes are collectively referred to as cell decision making and are fundamental for the survival of every organism, from bacteria to humans (Magyar et al., 2007). In particular, the development of different observable characteristics, like morphological traits or gene expression patterns, from an isogenic population defines a phenotypic decision making and it is exploited in a number of different contexts, such as stem cells differentiation (Morrison and Kimble, 2006), senescence (Oren, 2003), embryogenesis (Zernicka-Goetz et al., 2009), cancer progression and metastasis formation (Zhang et al., 2014).

Epithelial to mesenchymal transition (EMT) is a notable example of phenotypic cell decision making that consists in the transdifferentiation of epithelial in mesenchymal cells (Lamouille et al., 2014). While EMT is fundamental for the development of complex organisms (Nieto et al., 2016) and for wound healing (Margadant and Sonnenberg, 2009), it is also exploited in pathological settings, such as induction of pharmacoresistance (Pisco and Huang, 2015; Zheng et al., 2015), metastases formation (Margadant and Sonnenberg, 2009; Nieto et al., 2016) or fibrosis (Margadant and Sonnenberg, 2009). Indeed, increased survival capabilities, motility, and fibrogenesis are hallmarks of this transition, together with marked modifications in cell shape and expression profile (Zhang et al., 2014), Figure 1.

**Figure 1:**
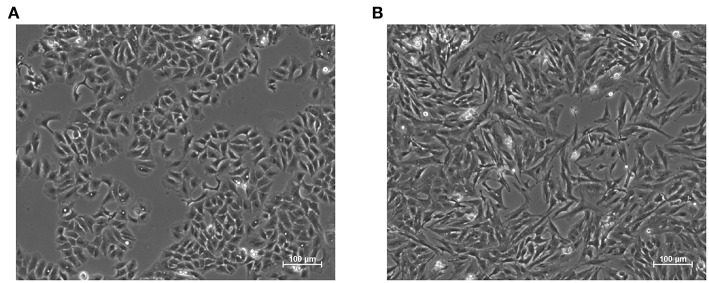
Representative images of the epithelial **(A)** and mesenchymal **(B)** phenotypes in A549 cell line. The latter was induced through treatment with the pro-fibrotic cytokine Transforming Growth Factor Beta 1, (TGF-β1, 5 ng/mL) for 48 h. Epithelial cells exhibit regular shapes and a high degree of spatial organization, while mesenchymal ones are more variable, spindle-like and have a less organized structure.

The importance of the EMT and its numerous biological roles have fostered a significant interest in the scientific community. In particular uncovering the underlying molecular mechanisms of this process is considered to be the key to its control and to the treatment and prevention of pathological processes. Traditional *in-vitro* assays, however, tend to be poorly equipped for this task, as they are designed to quantify population averages and are, therefore unable to evaluate intercellular variability. This parameter is crucial for complex biological processes, like phenotypic transitions, that initiate in a small subset of cells before spreading to the rest of the population. Computational strategies can be used to fill this gap, simulating the behavior of large networks and allowing to infer the response to complex stimuli (Andrieux et al., 2014), functionally clusterize the behavior of each cell (Coquet et al., 2017) or reconstruct developmental regulatory networks (Moignard et al., 2015). Most of these models, however require large amounts of quantitative data (Moignard et al., 2015) that might not be available, or specific information for every interaction, (Andrieux et al., 2014) that might restrict their applicability to a single experimental model.

Computational models can be exploited to identify the most relevant markers for a process of interest (Huang et al., 2013; Mak et al., 2016; Koplev et al., 2018) and drive its experimental evaluation. These gene lists, however, are generally extracted from experimental data and as such, tend to be highly dependent on the dataset composition. Furthermore, the gene selection methods often rely on the study of the correlation with the expression of putative EMT markers (seeds) (Mak et al., 2016; Koplev et al., 2018). As a consequence, they are not able to identify genes that are connected with non-linear relations to the seeds or that exhibit different gene expression trends in different cell lines. Furthermore, the currently available gene signatures include a high number of genes [33 in Huang et al. (2013), 77 in Mak et al. (2016), and 239 in Koplev et al. (2018)] that might limit their applicability and hamper the interpretation of the results.

In this study we describe a computational model of EMT that integrates a transcriptional regulation network and a discrete time Markov Chain (DTMC). This tool was developed to be independent from a specific cell line and particular *in-vitro* data. The signal transduction network was represented as a boolean network (BN) (Wang et al., 2012), that can be used to describe large networks and requires minimal information to be defined. The DTMC (Gagniuc, 2017) was automatically identified through the analysis of the stable states of the BN. This architecture allows both to study the temporal evolution of the different gene expression patterns (phenotypes) and to identify the most relevant nodes for the network's functioning. These markers could be used to characterize EMT progression and aid the interpretation of computational and experimental results.

Furthermore, this framework is not EMT specific and relies solely on publicly available information. As such, it could be effectively applied to other biological processes and become a general approach for combining *in-silico*/*in-vitro* study of complex cell population dynamics and identifying their most relevant drivers. To further improve applicability the code and the model are made freely available, both as Supplementary Material (Data Sheet 2) and at http://www.mcbeng.it/en/category/software.html

## 2. Materials and Methods

### 2.1. Definition of the Boolean Network (BN)

The BN was defined combining 24 signal transduction networks downloaded through Cytoscape (cytoscape.org) from the KEGG database (http://www.kegg.jp, Table S1). These maps were selected as the ones that included at least six markers commonly associated with EMT (Epithelial to Mesenchymal Transition RT^2^ Profiler PCR Array, Qiagen) and were independently converted in BNs, substituting each interaction type coded in KEGG with a boolean operation. These correspondences are reported in Table 1 where MAJ represents the majority function, that determines a gene to be ON if most of its inputs promote its activation, AND is the logical AND operation and the last two interactions types were not considered for the BN definition.

**Table 1:** Correspondences between Kegg interaction types and boolean operations used in the BN.

**Kegg interaction type**	**Boolean operation**
Activation	MAJ
Binding/association	AND
Complex	AND
Dissociation	AND
Expression	MAJ
Inhibition	MAJ
Missing interaction	-
Remove	-

This representation is a radical simplification with respect to the reality of gene expression regulation and might not be thoroughly accurate, but it is generally applicable to all the signal transduction pathways considered for this analysis. Indeed, using different logical functions or diversifying them would have required additional knowledge on each pathway, that could not be available or be dependent on the experimental model considered, thus reducing the applicability of this framework and complicating the integration of the different pathways.

The 24 logical networks extracted from the KEGG database, were combined in a single graph, relying on the Entrez gene ID, to uniquely identify each molecular component and effectively combine its interactions in different pathways (as exemplified in Figure S1). This resulted in a BN comprising 895 nodes and 171 connected components (i.e., independent subgraphs) identified using the algorithm proposed by Edelsbrunner and Harer (2010). The latter analysis highlighted the presence of a major subnetwork significantly larger (about 80 fold) than the others (Table S2), that was considered to comprise all the relevant regulations for the modeling of EMT in single cells.

The largest connected BN was analyzed to identify the most important nodes for the network's functioning. This step relied on an automatic and objective procedure that calculates a score based on two metrics commonly used for the analysis of BNs. The first one is the number of exiting connections, that quantifies the regulatory power of a gene through the evaluation of the number of nodes it influences directly. The second one is the eccentricity, that measures the centrality of each gene (Equation 1) as the inverse of the longest shortest distance between the current node and every other element of the network. Eccentricity can be considered an indication of the number of direct and indirect interactions of each node.

(1) $$\begin{array}{l} E=\frac{1}{max\left(minPathLen\right)} \end{array}$$

As each of these two parameters spans different ranges, the ranking ($R_{OD}^{G}$, $R_{E}^{G}$) of each gene/complex with respect to either parameter was used to calculate a score. This simple modification ensures an equal contribution of the two metrics to the score. While the application of the negative logarithm establishes a direct correlation between this parameter and the node's importance (Equation 2).

(2) $$\begin{array}{l} S_{G}=-log\left(R_{OD}^{G}+R_{E}^{G}\right) \end{array}$$

This metric, computed for every node of the BN, was used to isolate a small number of markers fundamental for the network's functioning, as modifications of their activities will extensively impact the network's dynamic behavior, and thus potentially capable of recapitulating EMT *in-vitro*.

### 2.2. Definition of the Discrete Time Markov Chain (DTMC)

A DTMC is defined by a set of states and a transition matrix, that identifies the connections between the nodes and their probability. Both these elements were determined automatically, through a local search algorithm (Algorithm 1, Figures S2A,B) capable of simulating the dynamic evolution of the state of all the nodes in the BN. In particular, starting from an initial condition defined as described in the next paragraph, the value of each gene/complex was updated according to its interactions with the rest of the network and the values of the other nodes. To simulate the presence of processes with different rates, the order with which the nodes were updated was defined randomly at each iteration (function defineUpdateOrder in Algorithm 1). This procedure is akin to asynchronous update but it constrains all the nodes to be revised within the same iteration (Figure S2C). This modification does not affect the single state attractors of the network and is expected to provide almost equivalent results in real-world networks.

**Algorithm 1:** DTMC Determination.

1: **procedure** DTMC-D(Network, PercActiveNodes, maxIter)
2: nodes ← number of nodes in the network
3: initialCondition ← defineInitialCondition(nodes/stable state, PercActiveNodes/PercPerturbation)
4: change ← 1 ⊳ flag used to track changes in the nodes configuration
5: iter ← 0 ⊳ index that keeps track of the number of iterations
6: newState ← initialCond
7: **while** change == 1 — iter < maxIter **do**
8: newState ← oldState
9: updateOrder ← defineUpdateOrder(nodes)
10: **for** o in updateOrder **do**
11: newState ← updateNode(newState,o)
12: end **for**
13: iter ← iter+1
14: **if** oldState==newState **then** ⊳ The configuration hasn't changed
15: change ←0
16: **end if**
17: **end while**
18: **return** newState
19: **end procedure**

A different determination of the initial condition was implemented for the identification of either the nodes and or the transition matrix of the DTMC. In the former case a random configuration with a defined probability of activation, varying between 0 and 100% in 5% steps, was used. In the latter a perturbed version of each stable state was considered. In particular the value of each gene was modified with a 5% probability, that could be increased in 5% steps in case the steady state reached at the end of the simulation was equal to the starting one. This procedure was iterated until either a new attractor was found or the flipping probability reached 100%. This strategy doesn't closely mirrors the behavior of a real population of cells, nor reproduces faithfully specific configurations recognizable *in-vitro* but allows for a much more extensive characterization of the dynamic behavior of the network while maintaining generality and user-independence.

A total of 80 · 10^4^ simulations were performed (40 · 10^4^ for the determination of the steady states and 40 · 10^4^) for the identification of the transition matrix. This number is significantly higher than that considered in similar works (Albert et al., 2008; Schwab et al., 2017) and it was chosen to account for the larger state space and maintain sampling accuracy. In both cases finding an attractor or reaching the maximum number of iterations (1 · 10^5^) was considered as a stopping criterion. During the determination of the transition matrix, a small number of simulations (about 10%) identified stable configurations that were not among the previously identified states of the DTMC. In these cases the end state was modified, so as to become equal to the node with the closest configuration for whom the edges has been determined. This modification was shown to always affect just one gene of the BN.

### 2.3. Simulation of the DTMC

The DTMC was simulated applying Equation 3 where s_*i*_, represents the fraction of the population exhibiting phenotype i, p_*jk*_ is the probability of transition from state j to state k, and t is the current iteration.

(3) $$\begin{array}{l} \left[\begin{array}{c} s_{1}\left(t+1\right)\\ s_{2}\left(t+1\right)\\ \vdots \\ s_{n}\left(t+1\right) \end{array}\right]=\left[\begin{array}{ccccc} p_{11} & p_{12} & p_{13} & \ldots & p_{1n}\\ p_{21} & p_{22} & p_{23} & \ldots & p_{2n}\\ \ldots & \ldots & \ldots & \ldots & \ldots \\ p_{n1} & p_{n2} & p_{n3} & \ldots & p_{nn} \end{array}\right]\cdot \left[\begin{array}{c} s_{1}\left(t\right)\\ s_{2}\left(t\right)\\ \vdots \\ s_{n}\left(t\right) \end{array}\right] \end{array}$$

The initial condition, that is the prevalence of each phenotype at the beginning of the simulation, was identified using publicly available RNA sequencing (RNA seq) data (Mishra et al., 2017) (available at https://www.ncbi.nlm.nih.gov/geo/ accession number GSE90566). In this study EMT is induced through the supplementation of 5 ng/mL of TGF-β1 for 72 h and its effects were evaluated in two cancer cell lines (a human non small cell lung cancer, A549 and a pancreas ductal adenocarcinoma, Panc1). These experimental results were here used to define the number of cells expressing each marker of the signature, that was considered to be proportional to the mean number of reads normalized by sequencing depth (baseMean). The gene characterized by the highest mRNA level (baseMean value) was assigned a random prevalence between 50 and 100%, determining the number of cells expressing every other marker as the fraction of this number maintaining the baseMean ratio. These thresholds were combined with an iterative procedure to identify 1,000 virtual populations of epithelial cancer cells undergoing TGF-β1-induced EMT, whose patterns of expression were coherent with the RNA seq data.

These populations of TGF-β1 expressing cells, that were simulated until steady state, and the results of this analysis were compared to experimental data acquired in comparable conditions, to validate the model. At the same time the DTMC was used to simulate single phenotypes and to determine their contribution to the macroscopic behavior observed *in-vitro*.

### 2.4. Cell Culture and Real-Time Quantitative PCR Data

In addition to the RNA Seq data presented in Mishra et al. (2017), the level of expression of the signature genes was also evaluated with real-time quantitative polymerase chain reaction (qPCR). These experiments were specifically performed to validate this computational framework inducing A549 cells with TGF-β1 (5 ng/mL for 72 h). A549 cells (American Type Culture Collection - ATCC, Manassas, VA, USA) were cultured in Dulbecco's Eagle's Modified Medium (DMEM, Pan-Biotech GmbH, Aidenbach, Germany) supplemented with 10% fetal bovine serum, 100 U/mL penicillin, 100 g/mL streptomycin and 2 mM L-glutamine (all from EUROCLONE S.p.A., Pero, MI, Italy), and maintained at 37°C in a humidified 5% CO_2_ incubator (Thermo Fisher, Waltham, MA, USA). Cells were seeded at a density of 24 × 10^4^ cells/cm^2^, 24 h prior to TGF-β1 (Peprotech, London, UK) administration.

To isolate total RNA, cells were washed with PBS and scraped. Cell pellets were then resuspended in 1mL of PureZOL^TM^ (Bio-Rad, Segrate, MI, Italy) and a standard protocol was applied. RNA was treated with DNAse I (Sigma-Aldrich, Milan, Italy) according to manufacturer instructions. 500 ng of total RNA was then retro-transcribed into cDNA with the iScript^TM^ cDNA Synthesis Kit (Bio-Rad, Segrate, MI, Italy) and diluted 1:10. cDNA was then amplified by qPCR using the SsoAdvanced^TM^ SYBRⓇ Green Supermix (Bio-Rad, Segrate, MI, Italy) and KiCqStartⓇ SYBRⓇ Green Primers (H_B2M_1, H_GAPDH_1, H_CDH1_1, H_VIM_1, H_COL1A1_2, H_EGFR_1, H_ITGB1_1, H_MAPK1_1, H_BAMBI_1, H_CBLC_1, H_FOXO6_1, and H_TLR2_1 from Sigma-Aldrich, Milan, Italy).

The CFX Connect^TM^ Real-Time PCR Detection System (Bio-Rad, Segrate, MI, Italy) was used to apply the following 2-step thermal-cycling protocol: 95^o^C for 3 min, followed by 40 cycles of 95^o^C for 15 s and 60^o^C for 30 s. A melting step between 95 and 65^o^C was also included. Data analysis was conducted using the CFX Manager^TM^ Software (Bio-Rad, Segrate, MI, Italy), creating a gene study that uses an inter-run calibrator to normalize the variability among the experiments. A total of three independent biological replicates performed in technical duplicates were performed.

## 3. Results

### 3.1. Definition of the Boolean Model

**Figure 2:**
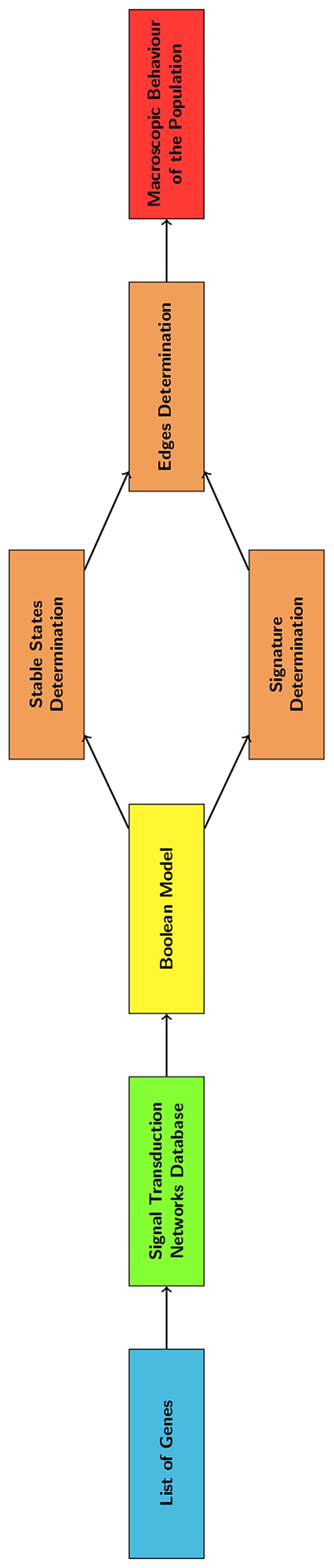
Flowchart describing the main steps of the computational method here described.

The computational model of EMT was defined using the hierarchical algorithm summarized in Figure 2. Its first step was the definition of a boolean model (BN) describing the relationships among the genes that are potentially involved in this phenotype transition. Boolean models associate a binary variable to each gene (node) and a Boolean operation to each connection (edge). In this case a logical AND was used to model the formation of complexes, thus associating an ‘on’ state with the presence of all the composing proteins. Gene regulation, on the other hand, was represented with the majority (MAJ) rule; i.e., a gene is set to one when most of its inputs induce activation.

This formalism was chosen because it requires a minimal set of information (only the logical relationships among the components) to be defined and, as a consequence, it can be generally applied to all the considered pathways, and to biological processes not entirely characterized. This characteristic is particularly relevant as introducing process-specific information (i.e., using different representations for the same kind of reaction in different pathways) might tie the BN to a specific experimental model or condition. Indeed, the purpose of this initial step was to include as much information as possible into the computational model and to provide a general description of the EMT in a wide range of experimental models and conditions. As such, the KEGG database (http://www.kegg.jp) was interrogated to identify, the signal transduction networks including a relevant number of EMT markers (details in Methods section). This database contains more than 500 representations of signal transduction pathways manually curated by experts, that describe the interactions among gene products (proteins and RNA) mostly independently of the experimental model.

These signal transduction networks were then combined into a BN (Figure S1) whose analysis revealed the presence of a main connected component, accounting for 78% of the nodes, and several other independent subgraphs of much smaller size (Table S2). As complex processes such as the EMT are characterized by the interaction of multiple regulatory mechanisms, we decided to disregard the smaller networks and focus on the major connected component of the BN, comprising 700 nodes and 1,364 edges.

### 3.2. Determination of the DTMC and of the Gene Signature

As shown in Figure 2, the BN was analyzed to determine the nodes and edges of the DTMC. This was achieved through the determination of the BN stable states and of the system's response to a perturbation of sufficient amplitude.

Attractors of length 1 have been previously associated with the phenotypes that a cell can assume (Kauffman, 1969a,b) and consequently to the nodes of a Markov chain describing the process of interest (Gupta et al., 2011; Chu et al., 2017). Concurrently a slight change in the expression profile of an attractor can be used to determine its connections to the other states of the DTMC and their probability.

To this aim, a local search algorithm (Algorithm 1) was implemented and used to identify the stable states with the largest basin of attraction. This strategy is particularly convenient for networks like the one considered here, that are characterized by a mostly unidirectional flow of information. This feature is likely to reduce cyclic attractors, since the number of feedbacks capable of leading the system to a previously visited configurations is very low. Additionally, this approach does not constrain the size of the network and allows for the simulation of processes with different temporal dynamics within the network.

The initial condition was set randomly and characterized by the probability of activation of each node that was varied between 0 and 100% with 5 % increments. This strategy was chosen for its generality and lack of hypotheses on the modeled system. Indeed setting specific initial conditions for the BN is likely to introduce constraints on its dynamic behavior, that might not be valid for all the experimental models of interest.

The number of stable states determined by this analysis, was of the same order of magnitude of the initial conditions. While this is to be expected, given the size of the network and the wide range of initial conditions, it prevents a meaningful analysis of the results and the determination of the population's behavior. For this reason an automatic and objective procedure to identify the most relevant nodes for the network's functioning was developed. This method relies on a score that combines two topological indices commonly used for the analysis of BNs (Equation 2) and was used to quantify the dependence of the paths within the network on the presence of each node.

**Figure 3:**
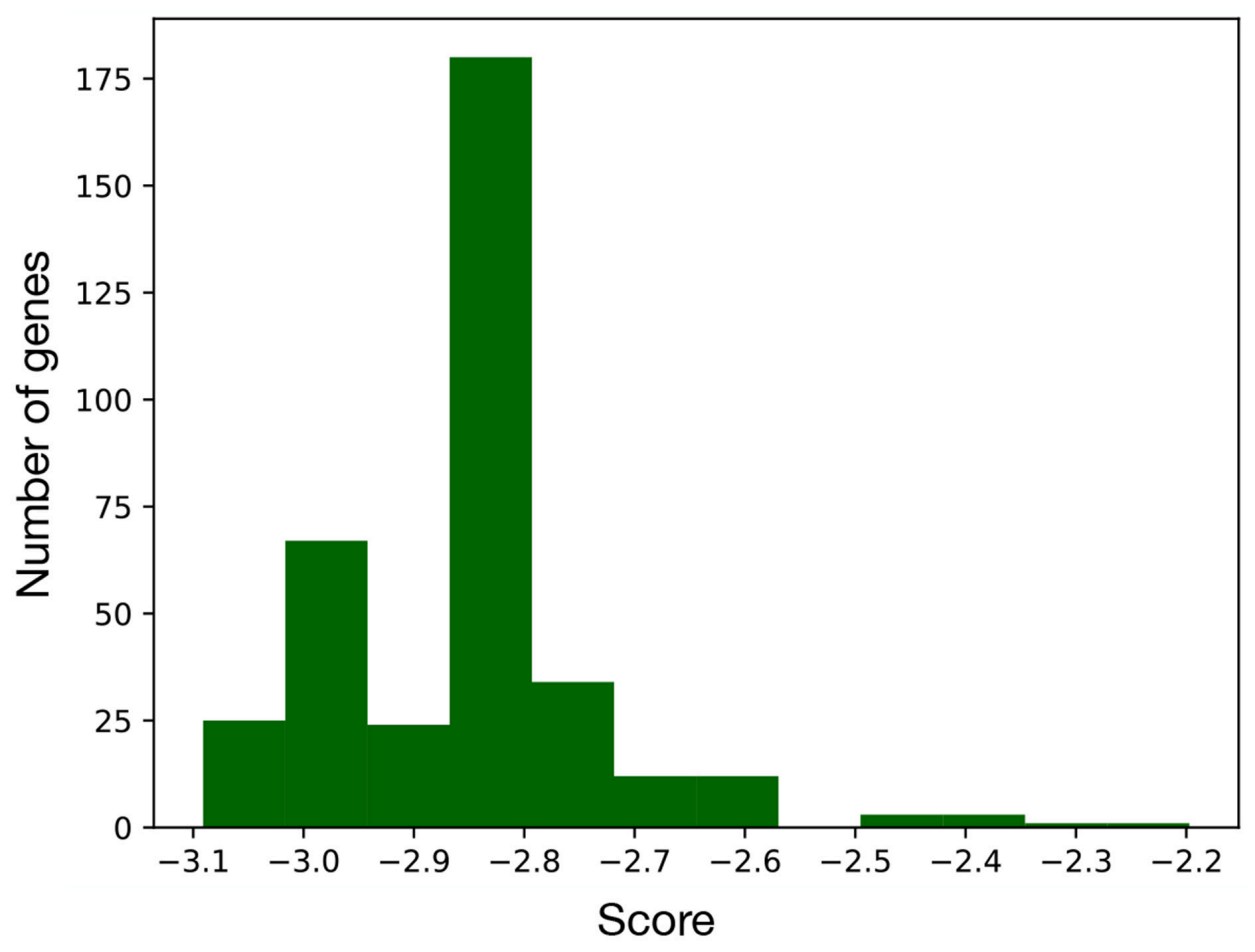
Distribution of the scores computed for every node of the EMT network. As this metric is proportional to the importance of the corresponding node, the genes with a score above –2.5 were considered as part of the signature.

Computing the index in Equation 2 for every node of the network led to the distribution of scores in Figure 3, that is characterized by two distinct populations. The former is the most numerous and comprises all the nodes with score below –2.5, while the latter includes the genes/complexes with the highest scores (above –2.5) and thus with the highest importance for the BN. These were considered as signature markers and are reported in Table 2, together with a short description of their function. Although most of them are not commonly reported EMT markers, their behavior is strictly connected to the hallmarks of this transition, such as proliferation, cancer progression, tissue repair, immune response, and differentiation.

**Table 2:** Signature genes determined to be significant for the behavior of the network and used to reduce the stable states.

**Gene**	**Note**
*BAMB1*	Pseudoreceptor of the TGFβ pathway whose function might be to limit the signaling range of the TGFβ family.
*CBLC*	Member of the Cbl family of E3 ubiquitin ligases that plays an important role in cell signaling. Its expression might be restricted to epithelial cells.
*EGFR*	Receptor for members of the epidermal growth factor family.
*ERBB2*	Member of the epidermal growth factor receptor family. While it does not have a ligand binding domain it acts as a stabilizer for the interaction between other receptors and ligands of the same family. Overexpression of this gene has been reported in numerous cancers.
*FOXO6*	Transcription factor, generally localized in the nucleus and elevant marker for the regulation of cancer development (e.g., lung and gastric cancer), whose effect has been shown to be context dependent (Jacobs et al., 2003; Qinyu et al., 2013; Hu et al., 2015).
*ITGB1*	Membrane receptor involved in cell adhesion and a variety of processes like embryogenesis, tissue repair, immune response and metastatic diffusion of tumor cells.
*MAPK1*	Kinase that integrates multiple biochemical signals, and is involved in a wide variety of cellular processes, such as proliferation, differentiation, transcription regulation and development.
*TLR2*	Receptor involved in pathogen recognition and activation of the innate immunity.

This list was completed with the addition of TGF-β1, widely used as an inducer to study the EMT *in-vitro*. The presence of this inducer was used to trigger the EMT in the DTMC (section 3.3).

The pattern of expression of the signature genes was used to merge the attractors previously determined for the BN into a small number of states (Figure S3). This step led to an almost 800-fold reduction in the number of stable configurations and the definition of the DTMC states shown in Figure S4 (total number of nodes 485).

Successively the transition matrix of the DTMC was determined through the simulation of the BN starting from a slightly perturbed version of each attractor. This perturbation, implemented by flipping the value of each gene with a defined probability, induces the transition between two stable states and thus identifies the connections among the phenotypes. As detailed in the material and methods section, this analysis, considered a starting flipping probability of 5%, that could be progressively increased, with 5% steps, if the perturbation did not trigger a transition toward another state.

After the simulation of all the fixed points, the probability of occurrence of each transition was determined as in Equation 4, where $N_{i}^{j}$ is the number of transitions recorded from state i to state j and N_*i*_ is the total number of transitions exiting state i.

(4) $$\begin{array}{l} P_{i}^{j}=\frac{N_{i}^{j}}{N_{i}}\cdot \frac{1}{d_{i}^{j}I_{j}^{i}} \end{array}$$

In this formulation the frequentist approach, that considers a uniform probability distribution for the transition rates, was combined with a correction factor aimed to increase the biological accuracy of the model. The relative frequency of each transition was divided by the distance between the two phenotypes ($d_{i}^{j}$) and the average length of the corresponding simulations ($I_{i}^{j}$). This operation favors the connections between similar stable states without imposing a threshold on the maximum number of genes that can vary at the same time. Concurrently, this correction increases the probability of transitions associated with shorter simulations, which have often been shown to be the most probable in a biological setting (Flöttmann et al., 2012). The results of this analysis defines the transition matrix of the DTMC, that were then used to infer the dynamics of gene expression patterns in a population of cells undergoing the EMT.

### 3.3. Model Validation

To ensure the effectiveness of the model in reproducing the EMT, the equivalence between its results and experimental data acquired in comparable conditions has been verified. To this aim the DTMC was used to simulate EMT in two cancer cell lines (A549 and Panc1). The results of the simulations were compared with RNA seq data originally presented in Mishra et al. (2017) and freely available at NCBI GEO (accession number GSE90566). Additionally qPCR data acquired *ad-hoc* were considered for the lung cancer cell line A549. The DTMC was simulated considering 2,000 virtual populations, initialized as described in the material and methods section and accounting for a significantly larger cardinality than a population generally employed for an *in-vitro* assays (~ 12.4 × 10^6^ cells for A549 and ~15.7 × 10^6^ cells for Panc1 cells). This analysis determined the changes in each phenotype prevalence throughout the EMT and thus the variations in the number of cells expressing each marker of the signature. These results were represented as the logarithm in base 2 of the fold change from the initial condition (FC, Equation 5), that is directly comparable with the corresponding experimental values and allows to easily distinguish between overexpressed (FC>0) and underexpressed markers (FC<0).

(5) $$\begin{array}{l} log_{2}FG_{g}=log_{2}\frac{RNA_{M}^{g}}{RNA_{E}^{g}} \end{array}$$

**Figure 4:**
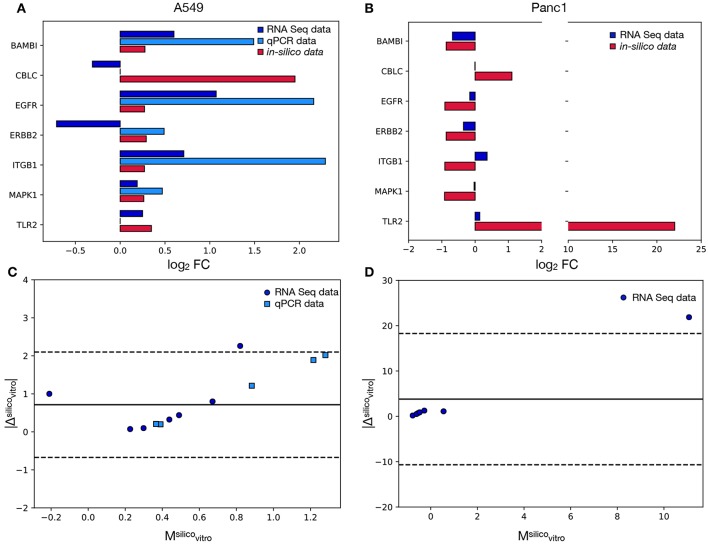
Comparison between the model's results and the *in-vitro* data. In **(A)** the log_2_FC recorded in A549 cells for each signature marker is reported both for the *in-vitro* and the *in-silico* analyses. Different colors are associated with alternative experimental techniques or the model's results. **(B)** is the same as in **(A)** but for the Panc1 cells. In this case only the RNA seq data reported in Mishra et al. (2017) were considered. **(C)** Bland-Altman plot showing the relation between the absolute difference of the *in-silico* and *in-vitro* data and their mean. Different colors and symbols identify different comparisons while the solid black line represents the average of the values on the y axis (obtained considering only the RNAseq values). The dotted lines identify the 95% limit of agreement (again computed using only the RNAseq data) and their distance from the solid line is 1.96·σ, where σ is the standard deviation of the absolute difference. **(D)** Same as in **(C)** but for the Panc1 cell line.

Figure 4 reports the results of this analysis and particularly panels A and B compare the FCs recorded *in-vitro* to the corresponding simulated values. One of the signature genes (*FOXO6*) was excluded from this analysis, since the corresponding mRNA level was not recorded in Mishra et al. (2017) and was below the qPCR's detection threshold. The latter technique was also unable to quantify the expression of *CBLC* and *TLR2*, that were however evaluated in Mishra et al. (2017). As such the comparison between the qPCR and the *in-silico* data was restricted to five markers (*BAMB1, CBLC, EGFR, ERBB2, ITGB1, MAPK1*), while for the RNAseq dataset all genes but *FOXO6* were considered.

The computational model shows a remarkably good agreement with the experimental data. In particular, the concordance index, calculated as the fraction of markers with the same variation sign between modeled and *in-vitro* data, is 100% for the qPCR and 71% for the RNA seq. This ability of the model of predicting the direction of change of the tested genes supports the efficacy of the proposed computational method, that works independently on the studied cell line. Furthermore the computational framework here presented was also able to predict, with the same DTMC, the direction of variation of markers that were shown to behave differently in the considered cell lines (e.g., *BAMB1, EGFR, MAPK1*). This is a very important characteristic of our model that greatly improves its range of applicability.

When the RNAseq dataset was considered, however, two markers for every experimental model were shown to behave incoherently *in-vitro* and *in-silico*. One of these, *CBLC*, was common to both cell lines while *ERBB2* and *ITGB1* were unique to A549 and Panc1 cells respectively.

A possible explanation for these inconsistencies is the non-linear relation between mRNA and protein levels. Indeed the pathways in the Kegg database describe high level interactions involving mostly proteins, while the experimental data used for the validation of the model quantify mRNA. As post-transcriptional gene expression regulation mechanisms and mRNA degradation rates could disrupt the proportionality between the level of transcript and the amount of active protein present in the system, a small percentage of inconsistently expressed genes is to be expected.

The discrepancy between the qPCR and RNA levels of *ERBB2* (Figure 4A) on the other hand, could be caused by the low basal expression level of this gene. Indeed in this condition, stochasticity of gene expression and population composition could induce important changes in the FC.

Despite these differences the *in-silico* results were shown to be equivalent to the qPCR results and to have a very good agreement with the RNA seq ones. Indeed all the data points obtained with the former technique were localized within the 95% limit of agreement (Equation 6) of the respective Bland Altman plots (dotted lines in Figure 4C), while only one marker for every cell line (corresponding to *CBLC* for the A549 and *TLR2* for the Panc1) was outside of the 95% confidence interval when the RNA seq was considered (Figures 4C,D).

(6) $$\begin{array}{l} LoA=|\bar{\Delta_{vitro}^{silico}}|\pm 1.96\cdot \sigma_{vitro}^{silico} \end{array}$$

This analysis determined also the presence of an offset, as the solid line corresponding to the average of the values on the y axis is above 0. This fixed error can almost entirely be attributed to the outliers (*CBLC* for A549 cells and *TLR2* for Panc1 cells), as all the other genes are characterized by significantly lower values.

The equivalence of the results obtained for the two considered cell lines, highlights once more the independency of our computational framework from the experimental model, and thus its versatility and usefulness for the study of complex biological processes.

### 3.4. Transient State Analysis

The final step of the analysis of the EMT model was the study of the transient state, that is the evaluation of the different paths that EMT can follow and of their dependence on the initial configuration.

For this analysis the prototypal mesenchymal configuration, determined setting to 1 the markers upregulated by TGF-β1 induction and to 0 those downregulated by this stimulus (Mishra et al., 2017) (Table 3), was used as a reference point in the calculation of the Jaccard-Needham distance.

**Table 3:** Theoretical mesenchymal states determined for the A549 and Panc1 cell lines.

	**A549**	**PANC1**
*BAMB1*	1	0
*CBLC*	0	0
*EGFR*	1	0
*ERBB2*	0	0
*ITGB1*	1	1
*MAPK1*	1	0
*TLR2*	1	1

*Upregulation and downregulation, as measured by Mishra et al. (2017) upon TGF-β1 stimulation, correspond to 1s and 0s, respectively*.

This parameter was computed as in Equation 7, where TF and TT are the numbers of genes that are respectively set to 0 and 1 in both configurations, while FT is the number of markers that are 1 in the test configuration and 0 in the reference one.

(7) $$\begin{array}{l} JND=\frac{TF+FT}{TT+TF+FT} \end{array}$$

The temporal evolution of the average value of this parameter, weighted on each phenotype prevalence, was used to track the state of the simulation and summarize its distance from the theoretical EMT endpoint.

**Figure 5:**
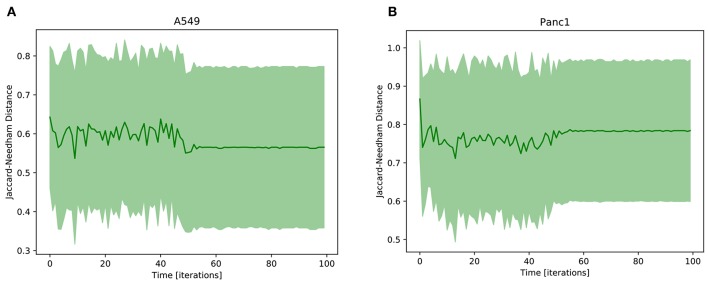
Average Jaccard-Needham distance between the 10% most expressed phenotypes and the theoretical mesenchymal state (Table 3). The error bars represent the standard error. In **(A)** the results of A549 cells are reported, while **(B)** shows the Panc1 data.

The result of this analysis is reported in Figure 5, where the Jaccard-Needham distance from the prototypal mesenchymal configuration is shown to slightly decrease and stabilize throughout the simulation. This is an indicator of a progression of the phenotypic transition that is however associated with a significant variability throughout its execution. This is probably a consequence of the many different paths that cells undergoing EMT can follow. Indeed as shown in Flöttmann et al. (2012); Wang et al. (2014) phenotypic transitions do not proceed linearly from start to end through the same set of states, but are composed of several different routes that can be influenced and determined by many factors, including gene expression stochasticity, variability in the microenvironment, differences in cell cycle phase and epigenetic regulation. This aspect complicates the analysis of biological processes, as population averages become unsuited for the study of their intermediate phases. Computational tools can be particularly helpful in this regard, since they allow to follow the behavior of each uniform subpopulation independently.

The *in-silico* model of EMT was then used to simulate cell populations composed of a single TGF-β1-expressing phenotype and determine the path associated with their transformation and the corresponding effectiveness. The latter was quantified as the variation, between the first and last iteration, in average Jaccard-Needham (Jaccard, 1901) distance from the theoretical mesenchymal state (the same used for the A549 cell line), weighted on the prevalence of each phenotype.

Of the 234 TGF-β1 expressing phenotypes, 8 were associated with a high transformation efficiency, defined as a difference in the Jaccard-Needham distance above 40%. The analysis of the expression pattern of these states led to the identification of 4 couples of phenotypes that differ only for the value of *FOXO6* (Table 4).

**Table 4:** Expression patterns of the phenotypes associated with the highest EMT efficiency.

**Couple**	**Phenotype**	**TGFβ1**	***BAMB1***	***CBLC***	***EGFR***	***ERBB2***	***FOXO6***	***ITGB1***	***MAPK1***	***TLR2***
1	7	1	0	0	0	0	0	0	0	0
	248	1	0	0	0	0	1	0	0	0
2	13	1	0	1	0	0	0	0	0	0
	291	1	0	1	0	0	1	0	0	0
3	24	1	0	0	0	1	0	0	0	0
	348	1	0	0	0	1	1	0	0	0
4	77	1	0	1	0	1	0	0	0	0
	343	1	0	1	0	1	1	0	0	0

**Figure 6:**
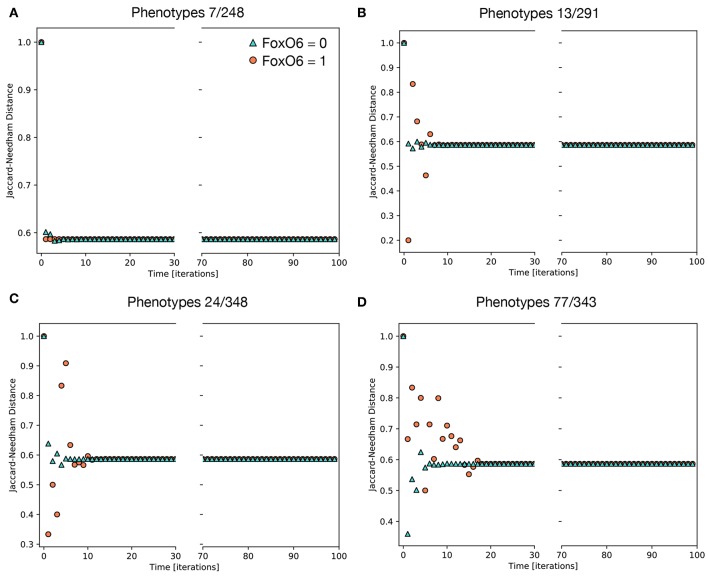
Average Jaccard-Needham distance between the theoretical mesenchymal state and the populations generated through the simulations of single phenotypes. The eight initial configurations associated with the largest variation of this parameter are shown and divided in 4 couples differing only for the value of *FOXO6* (Table 3). The traces in **(A)** describe the behavior of the first couple, phenotypes 7 and 248, while **(B–D)** refer to couples 2, 3, and 4, respectively. In all cases cyan triangles indicate the results obtained in absence of *FOXO6*, while the orange circles represent the condition of active *FOXO6*.

Their behavior is reported in Figure 6, where the effect of the activation of *FOXO6* is highlighted. In particular, the phenotypes not expressing this marker are associated with a short transient state and little or no oscillations, while the activation of this gene, combined with the expression of either *CBLC* (Figure 6B) or *ERBB2* (Figure 6C), generates important non-monotonic variations of the Jaccard-Needham distance and a consistent increase in the number of iterations required to reach the steady state. The expression of The expression of both *CBLC* and *ERBB2* (Figure 6D) is associated with an enhancement of this phenomenon that appears also when *FOXO6* is off, albeit with a shorter dynamic.

Even though *FOXO6* is a poorly studied marker within the EMT framework, partly due to its low expression level in the cellular models commonly used to study this phenomenon (i.e., TGF-β1-induced A549 cells), this computational analysis suggests it might have an important role in increasing the variability among the population and generating the different EMT paths. Its contribution, however, is potentially difficult to verify *in-vitro*, as it requires the identification of the subpopulations of phenotypes and thus the evaluation of gene expression patterns at single cell level.

## 4. Discussion

This work presents a computational model of EMT, a biological process that enhances the invasiveness of epithelial cells and has thus been associated to cancer progression and metastasis formation. The complexity of this phenomenon, that features a large regulatory network and can proceed through a number of different pathways, has hampered its understanding and characterization through *in-vitro* assays. An *in-silico* study, however, can overcome the limitations of the experimental analysis and both consider large signal transduction networks and determine the role of single regulatory units. Indeed while computational models represent an approximation of the biological reality, their results could be used to direct the experimental analysis and identify potential therapeutic targets.

In this study, specifically, a computational representation of EMT has been presented. It comprises both a single cell and a population level representations that were developed to be generally applicable independently of the experimental model. This choice might reduce the biological accuracy of the model, as it disregards the differences among cell lines and radically simplifies the regulation of gene expression. The presented tool, however, was shown to be able of automatically identifying a small set of regulatory keypoints whose patterns of expression could recapitulate EMT progression. The latter was measured as the average Jaccard-Needham distance from the prototypical mesenchymal phenotype. This composite parameter, being directly comparable with experimental data, was used to validate the computational framework, through the comparison of its results with both qPCR and RNA seq data. In both these cases the model was able to reproduce accurately the experimental results and was thus used to study how the different initial configurations contribute to the generation of the macroscopic behavior. This analysis identified 4 couples of phenotypes associated with a decrease in the average Jaccard-Needham distance from the mesenchymal state higher than 40% and differing only for the expression of *FOXO6*. The comparison of the paths followed by each element of the couple allowed to determine the effect of this marker in EMT progression. The activation of *FOXO6*, in particular, was shown to induce an increase in phenotypic variability within the population that resulted in oscillations in the average Jaccard-Needham distance and a longer transient phase. *FOXO6* is a transcription factor mostly found in the nucleus (Jacobs et al., 2003; Link, 2019) that has been recently linked to EMT as its silencing has been associated with reduced cell proliferation, migration and invasion in colon cancer cells, via the inhibition of PI3K/Akt/mTOR pathway (Li et al., 2018). The role of this gene, however, has been scarcely studied within EMT, partially due to its low average expression in common experimental models of this phenomenon. Furthermore it is worth noting that mRNA levels are not always an indicator of protein activation, as translation, post-translational modifications and protein localization can affect this property. Finally the activation of *FOXO6* was here shown to increase inter-cellular variability, an aspect that might be masked when evaluating population averages.

Population variability is emerging as a fundamental variable for the analysis and interpretation of biological processes, whose importance has been largely understated due to the technical difficulties in its experimental evaluation. The integration of *in-silico* and *in-vitro* approaches could be an effective solution to bridge this gap and develop accurate, quantitative methods for the evaluation of complex cellular phenomena at single cell level. The method here proposed is not EMT specific, relies solely on freely available information and is user independent and highly automated. As such its could potentially become a general approach for the integration of computational and experimental methods and the identification of the most relevant drivers of complex cell population dynamics.

## Author Contributions

MC developed the computational model, analyzed its results and compared them to the *in-vitro* data. AP conducted the experimental analysis (qPCR analysis). MC, SF, and EG contributed to the design and conception of the study. MC drafted the manuscript. All authors contributed to manuscript revision, read, and approved the submitted version.

### Conflict of Interest Statement

The authors declare that the research was conducted in the absence of any commercial or financial relationships that could be construed as a potential conflict of interest.
