# Supplementary material for: Identification via Numerical Computation of Transcriptional Determinants of a Cell Phenotype Decision Making
Source: Front Genet. 2019 Jun 21;10:575. doi: 10.3389/fgene.2019.00575 (PMC6598594; doi:10.3389/fgene.2019.00575)
Supplement: Supplementary file 1 [file Data_Sheet_1.PDF]

Supplementary Material:
Article Title

1 SUPPLEMENTARY TABLES AND FIGURES

1.1 Tables

**Pathways**	**Number of EMT Markers**
Proteoglycans in Cancer	24
Pathways in Cancer	23
Focal Adhesion	16
PI3K-AKT Signaling Pathway, Hippo Signaling Pathway	14
HTLV-I Infection	12
Signaling Pathway Regulating Pluripotency of Stem Cells	10
Regulation of Actin Cytoskeleton, Bacterial Invasion of Epithelial Cells, Leukocytes, Transendothelial Migration, Colorectal Cancer	9
Adherence Junctions, Amoebiasis, Pancreatic Cancer	8
WNT Signaling Pathway, RAP1 Signaling Pathway, MAPK Signaling Pathway, FoxO Signaling Pathway	7
Hepatitis B, Chagas Disease, Endometrial Cancer,Basal Cell Carcinoma, Toxoplasmosis, TGFβ Signaling Pathway	6
Platelet Activation, Small Cell Lung Cancer, RAS Signaling Pathway, Chemokine Signaling Pathway, Thyroid Hormone Signaling Pathway, Estrogen Signaling Pathway, Transcriptional Misregulation in Cancer, HIF-1 Signaling Pathway, Prostate Cancer, Melanoma, Osteoclast Differentiation, Renal Cell Carcinoma, Glioma, Inflammatory Bowel Disease (IBD)	5
Phagosome, Bladder Cancer, TNF Signaling Pathway, Rheumatoid Arthritis, Thyroid Cancer, Pathogenic Escherichia coli Infection, Graft-versus-Host Disease, Tight Junction, Hepatitis C, ErbB Signaling Pathway, Tubercolosis, Chronic Myeloid Leukemia, Choline Metabolism in Cancer, Cell Cycle, Leishmaniasis	4
Proteins Digestion and Absorption, Ehler-Danios Syndrome (EDS), Shigellosis, Penile cancer, Measles, Acute Myeloid Leukemia, Epstein-Barr Virus Infection, Hedgehog Signaling Pathway, Gastric Cancer, Toll-like Receptor Signaling Pathway, VEGF Signaling Pathway, Prolactin Signaling Pathway, Non-Alcoholic Fatty Liver Disease (NAFLD), Central Carbon Metabolism in Cancer, Calcium Signaling Pathway, Epithelial Cell Signaling in Helicobacter Pylori Infection, Malaria, Allograft Rejection, Axon Guidance	3

Cholinergic Synapse, Dopaminergic Synapse, Pertussis, GnRH Signaling Pathway, Choriocarcinoma, Waardenburg Syndrome (WS), Hepatocellular Carcinoma, Epidermolysis Bullosa Simplex, CGMP-PKG Signaling Pathway, Oxytocin Signaling Pathway, cAMP signaling Pathway, Sphingolipid Signaling Pathway, Jak-STAT Signaling Pathway, B Cell Receptor Signaling Pathway, Fc Epsilon RI Signaling Pathway, Fc Gamma R-Mediated Phagocytosis, Neutrophin Signaling Pathway, Adipocytokines Signaling Pathway, Non Small Cell Lung Cancer, Notch Signaling Pathway, Dorso-Ventral Axis Formation, Viral Carcinogenesis, Gap Junctions, Oral Cancer	2
Vascular Smooth Muscle Contraction, Osteogenesis Imperfecta, Glomerulopathy with Fibronectin Deposits (GFND), Lymphedemas, Circadian Entrainment, Retrograde Endocannabinoid Signaling, Glutamatergic Synapse, Serotonergic Synapse, GABAergic Synapse, Morphine Addiction, Alcoholism, Hematopoietic Cell Lineage, Torg-Winchester Syndrome, Metaphyseal Dysplasias, p53 Signaling Pathway, Complement and Coagulation Cascades, Inherited Thrombophilia, Plasminogen Activator Inhibitor Type 1 (PAI-1) Deficiency 46, XX Disorders of Sex Development, Mineral Absorption, Type II Diabetes Mellitus, Craniosynostosis, Vitroretinal Degeneration, Cataract, Core Neurocanthocytosis Syndrome, Choreoacanthocytosis, Breast Cancer, Nasopharyngeal Cancer, Naxos Disease and Carvajal Syndrome, Striate Palmoplantar Keratoderma, Skin fragility-Wolly Hair Syndrome, Interleukin 1 Receptor Antagonist Deficiency (DIRA), Malignant Melanoma, Ocular Albinism, Tietz Syndrome, Band-like Calcification with Simplified Gyration and Polymicrogyria (BLC-PMG), mTOR Signaling Pathway, AMPK Signaling Pathway, Apoptosis, Adrenergic Signaling in Cardiomyocytes, T Cell Receptor Signaling Pathway, Insulin Signaling Pathway, Progesterone Mediated Oocyte Maturation, Glucagon Signaling Pathway, Regulation of Lipolysis in Adipocytes, Carbohydrates Digestion and Absorption, Influenza A, PTEN Hamartoma Tumor Syndrome (PHTS), Autosomal Dominant Mental Retardation, Pilomatricoma, Type I Diabetes Mellitus, Letal Congenital Contractural Syndrome, Tetralogy of Fallot, Alagille Syndrome, Naegeli-Franceschetti-Jadassohn Syndrome, Dermatopathia Pigmentosa Reticularis, Heterotaxy, Prion Disease, Acute Lymphoblastic Leukemia, Bicuspid Aortic Valve, Loeys-Dietz Syndrome, Ossification of the Posterior Longitudinal Ligament of Spine (OPLL), Isolated Orofacial Clefts, Rienhoff Syndrome, Natural Killer Cell Mediated Cytotoxicity, Pancreatic Secretion, Amyotrophic Lateral Sclerosis (ALS), Salmonella Infection, Viral Myocarditis, Intestinal Immune Network for Iga Production, Camurati-Engelmann Disease, Esophageal Cancer, Cervical Cancer, Laryngeal Cancer, PPAR Signaling Pathway, Fuchs Corneal Dystrophy (FECD), Posterior Polymorphous Corneal Dystrophy (PPCD), l-Defined Immunodeficiency Syndromes, Endocrine and Other Factor-Regulated Calcium Reabsorption, Mowat-Wilson Syndrome	1

Table S1: Pathways of the Kegg database (http://www.kegg.jp) that contain the marker commonly associated to EMT. The ones including at least 6 markers were used to build the boolean EMT model.

Number of Nodes	Number of Connected Components
700	1
9	1
4	2
3	4
2	3
1	160

Table S2 Connected components that were determined applying the process described in Edelsbrunner *et al.* to the boolean network describing EMT determined using the information coded in the kegg (http://www.kegg.jp) database.

1.2 Figures

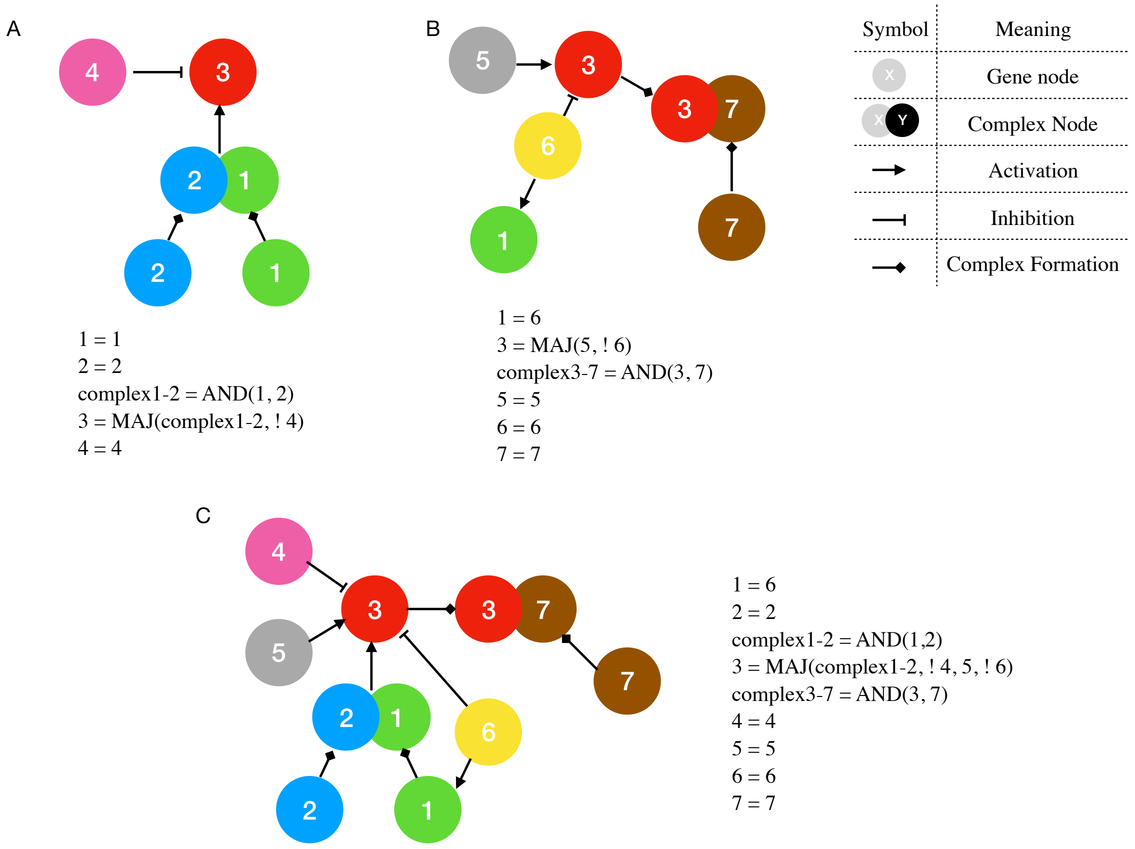

Figure S1. Schematic representation of the process employed to transform the Kegg pathways in boolean networks (BNs). Panels A and B report two simplified examples Kegg pathways and of their representation as BNs. While panel C shows the results of the operation both as network and as BN.

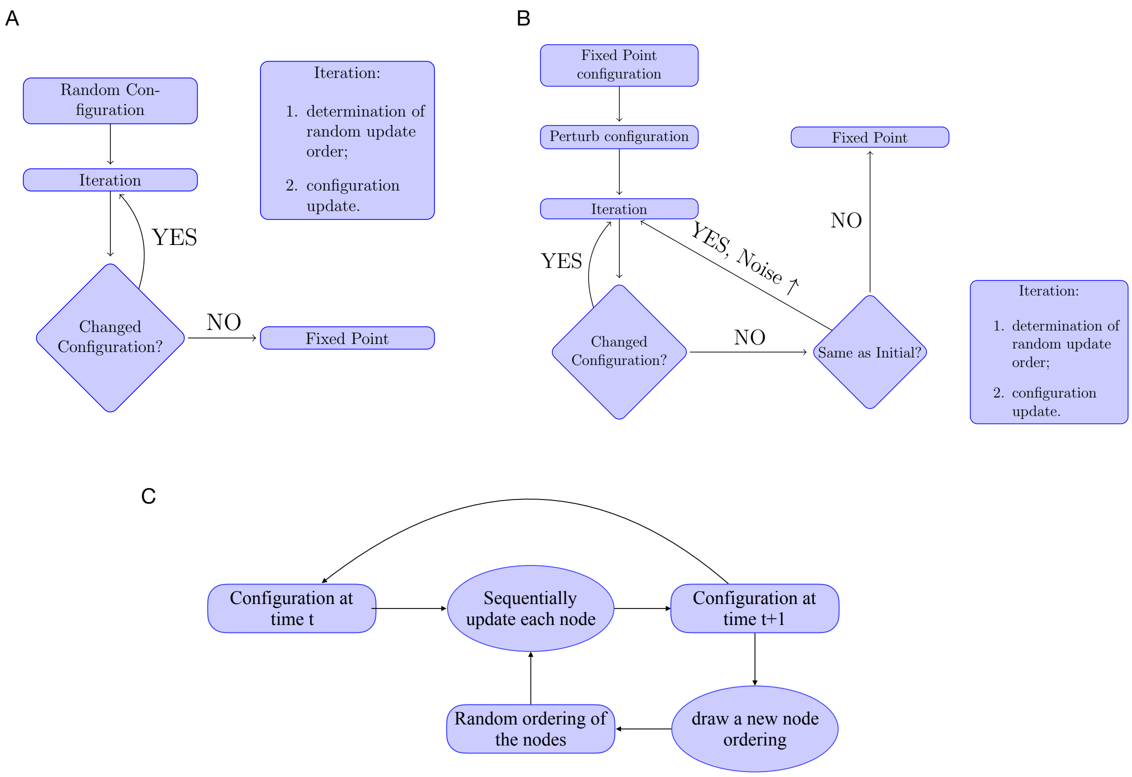

Figure S2. Flowcharts describing the main steps of the two versions of Algorithm 1. A shows the procedure applied to determine the nodes of the discrete time Markov chain (DTMC) while B represents the main steps taken to identify the transition matrix of the DTMC.

	Gene 1	Gene 2	Gene 3	Gene 4	Gene 5
Attractor 1	0	0	0	0	0
Attractor 2	1	0	0	1	1
Attractor 3	0	1	1	1	1
Attractor 4	1	1	1	0	0
Attractor 5	0	1	0	0	0
Attractor 6	0	1	1	1	1

	Gene 1	Gene 4	Gene 5
Attractor 1/5	0	0	0
Attractor 2	1	1	1
Attractor 3/6	0	1	1
Attractor 4	1	0	0

Figure S3. Schematic representation of the approach used to condense the attractors according to the values of the signature nodes. In this example genes 1, 4 and 5 compose the signature thus making attractors 1(3) and 5(6) equivalent in their reduced version.

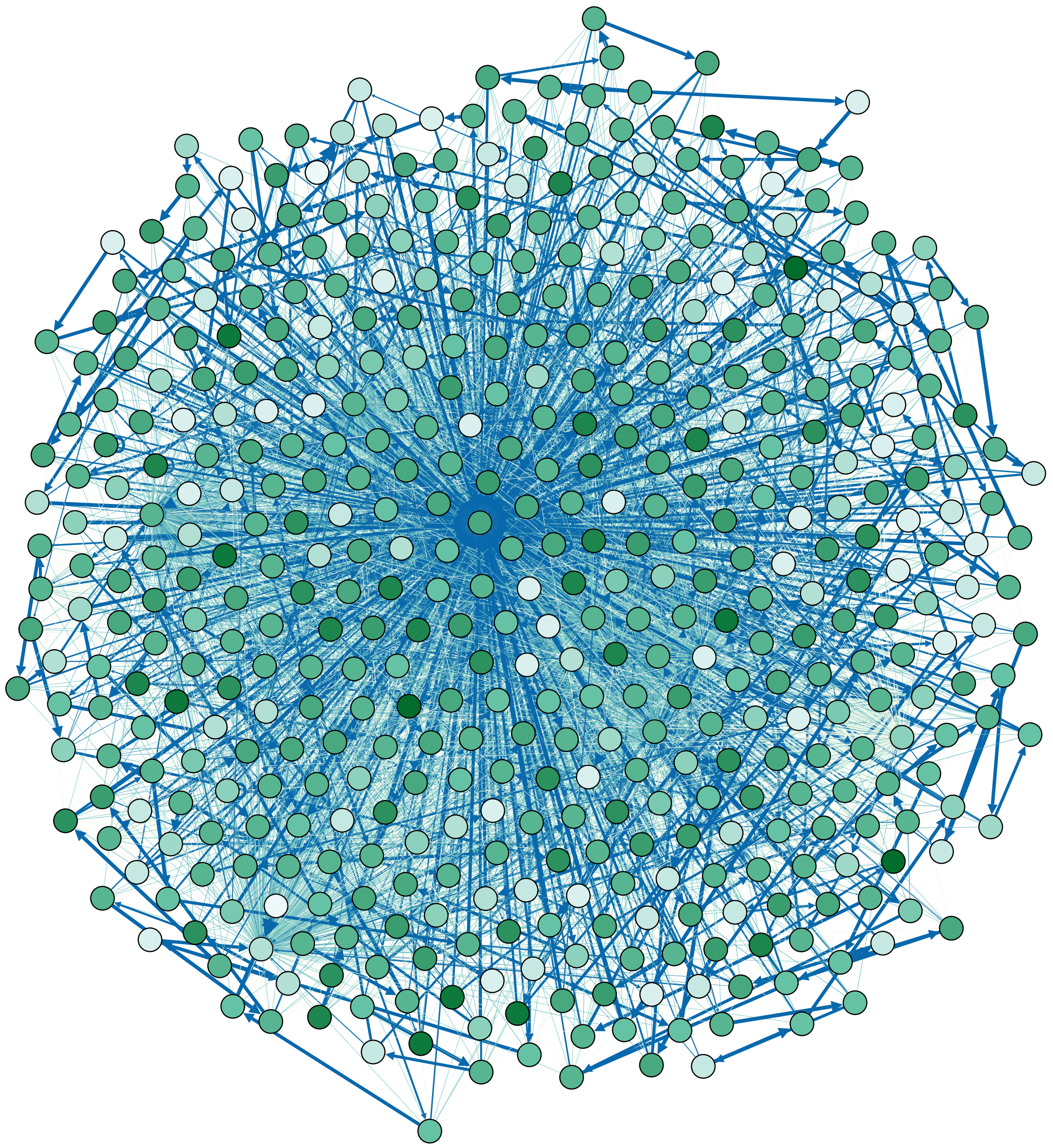

Figure S4. Graphical representation of the discrete time Markov chain (DTMC) used in this analysis. The intensity of the color of the nodes is proportional to the number of its exiting connections while the thickness of the edge represents the weight of the interaction.
