# Supplementary material for: Identification via Numerical Computation of Transcriptional Determinants of a Cell Phenotype Decision Making
Source: Front Genet. 2019 Jun 21;10:575. doi: 10.3389/fgene.2019.00575 (PMC6598594; doi:10.3389/fgene.2019.00575)
Supplement: Supplementary file 2 [file Data_Sheet_2.ZIP › ServerFolder/UserGuide.pdf]

Dear User,
please find in the following a minimalistic guide to the collection of scripts in this folder. They have been collectively used to create a computational representation of EMT that represents single cells with a Boolean Network (BN) and entire populations as Markov Chains (MC) and effectively reproduced the experimental behaviour of multiple cell lines, beside being able to isolate a small number of markers of potential therapeutic relevance (Cortesi et al. Identification via numerical computation of transcriptional determinants of a cell phenotype decision making).
The accompanying code is provided as is under the GNU public license (v2). Further questions of doubts can be directed to Marilisa Cortesi, PhD. (marilisa.cortesi2@unibo.it)

1. Download the Kegg Pathways of interest through Cytoscape (https://cytoscape.org) and export in .csv. This results, for each pathway, in two files named Edges.csv and Nodes.csv.

2. Run **1-cyto2Bool.py** to convert each pathway in a boolean network (BN). In the file update the value of the variable folder (line 8) with the path of the master folder containing one folder for each pathway (each containing the corresponding files Edges.csv and Nodes.csv). The execution of this file produces a .txt file (BNetwork.txt) containing the BN corresponding to each pathway.

3. Run **2-combineNets.py** that read all the BNs created with 1-cyto2Bool.py and combines them. In the file update the value of mainFolder (line 77) with the path of the master folder (the same as before) and fileOut (line 78) with the path and name of the output file.

4. Run **3-connectedComponents.py** to identify the connected components of the network. Update the values of file (line 41) with the path of the combined network (the output of 2-combineNets.py) and file2 (line 42) with the path of the output file. This will contain the list of genes (specifically their ID) comprised in each connected component.

5. Run **4-simplifyNet.py** to combine the results of 2-combineNets.py and 3-connectedComponents.py and create one text file (NetCC_*.txt) for each connected component containing the formalization of the corresponding BN. Update the values of variables fileCC (line 35), fileNet (line 36) folderOut (line 37) with, respectively, the path of the output of 3-connectedComponents.py, that of 2-combineNets.py and the path of the output folder.

6. Run **5-fixedPoints.c** to simulate the BN and identify its stable states. Update the variables from line 442 to 446 with the appropriate values (Table 1)

Line	Variable	Value
**442**	fileName	network to simulate (one of the outputs of 4-simplifyNet.py)
**443**	outFile	name of the output file
**444**	nodes	number of nodes in the network
**445**	initConds	number of initial conditions (i.e. simulations)
**446**	percActive	percentage of active nodes in the network

Table 1: variables to update in 5-fixedPoints.c

7. Run **6-MCEdges.c** to simulate the BN and identify the edges of the Markov chain (MC). Update the variables from line 566 to 574 as described in Table 2.

Line	Variable	Value
**566**	fileName	network to simulate (one of the outputs of 4-simplifyNet.py)
**567**	percActive	percentage of active nodes in the network
**568**	nFiles	number of output files from fixedPoints.c
**571**	nodes	number of nodes in the network.
**572**	initConds	number of initial conditions (for each output file from fixedPoints.c)
**573**	startNoise	initial perturbation (percentage)
**574**	noise	percentage step for increasing node perturbation

Table 2: variables to update in 6-MCEdges.c

8. Run **7-computeTopologicalParameters.py** to calculate, for every node of the network, the following topological parameters: in degree, out degree, eccentricity, closeness, eigenvectors. Update the value of the variable fileName (line 189) and outFile (line 182) with the paths of the BN and output file respectively.

9. Run **8-selectGenes.py** to compute the score (from the output of 7-computeTopologicalParameters.py) and select the signature. Update the values of fileIn (line 145) with the name and path of the output of 7-computeTopologicalParameters.py and fileOut (line 146) with the name and path of the output file.

10. Run **9-findEdges.py** to condense the attractors according to the values of the signature genes. Please update the values of the variables at lines 181, 182, 183 and 184 before proceeding. The first one is the path of the master folder containing the results of 6-MCEdges.c (containing one folder for each attractor each containing the corresponding files). The second is the BN (obtained with 4-simplifyNet.py) and the third is the list of the signature genes (obtained with 8-selectGenes.py). The last parameter is the path and name of the output file.

11. Run **10-defineMC.py** to produce two txt files containing the nodes and the transition matrix of the MC. Before launching the script initialize fileData (line 255) with the output of 9-findEdges.py, folderOut (line 256) with the path for the output files and selectedFile (line 257) with the file containing the signature genes.

12. Run **11-simulateMC.py** to simulate the MC. Set the variables at lines 214-222 (Table 3) before launching the script.

Line	Variable	Value
**214**	fileNodes	file Nodes.txt produced by 10-defineMC.py
**215**	fileTM	file Edges.txt produced by 10-defineMC.py

Line	Variable	Value
216	fileRNA	file containing, for each gene of the signature, its normalized prevalence in the population (an example below *)
217	folderOut	path where to save the outputs
218	maxTime	number of iterations
219	cellLine	name identifying the cell line
220	population	population cardinality
221	simulations	number of simulations
222	fixedNodes	dictionary containing the value of nodes to be kept fixed during the simulation.

```
*Cell Line:     Panc1
 Condition:     control
 Gene Expression[%]
 CBLC      0.00239005101431 +/- 0.000650489493809
 ERBB2     0.645579335088 +/- 0.0342316555232
 EGFR      2.40465688162 +/- 0.374443774004
 VIM       100.0 +/- 2.27967663352
 TLR2      0.0 +/- 0.0
 BAMBI     0.0156681122049 +/- 0.00590239317691
 MAPK1     1.97683775006 +/- 0.22013215463
 ITGB1     20.89010811 +/- 1.72853631263
 COL1A1    1.6828614753 +/- 0.182306958124
 CDH1      0.163320152645 +/- 0.0280314935733
```
